# A phase I study of the safety and pharmacokinetics of the histone deacetylase inhibitor belinostat administered in combination with carboplatin and/or paclitaxel in patients with solid tumours

**DOI:** 10.1038/sj.bjc.6605726

**Published:** 2010-06-15

**Authors:** U Lassen, L R Molife, M Sorensen, S-A Engelholm, L Vidal, R Sinha, R T Penson, P Buhl-Jensen, E Crowley, J Tjornelund, P Knoblauch, J S de Bono

**Affiliations:** 1 Department of Oncology, University Hospital, Rigshospitalet, Copenhagen 2100, Denmark; 2 Drug Development Unit, Institute of Cancer Research, The Royal Marsden Hospital, Sutton, Surrey SM2 5PT, UK; 3 Department of Haematology/Oncology, Massachusetts General Hospital, 55 Fruit Street, Boston, MA 02114, USA; 4 TopoTaget A/S, Symbion Science Park, Fruebjergvej 3, Copenhagen 2100, Denmark

**Keywords:** HDAC, belinostat, carboplatin, paclitaxel, BelCaP

## Abstract

**Background:**

This phase I study assessed the maximum tolerated dose, dose-limiting toxicity (DLT) and pharmacokinetics of belinostat with carboplatin and paclitaxel and the anti-tumour activity of the combination in solid tumours.

**Methods:**

Cohorts of three to six patients were treated with escalating doses of belinostat administered intravenously once daily, days 1–5 q21 days; on day 3, carboplatin (area under the curve (AUC) 5) and/or paclitaxel (175 mg m^−2^) were administered 2–3 h after the end of the belinostat infusion.

**Results:**

In all 23 patients received 600–1000 mg m^−2^ per day of belinostat with carboplatin and/or paclitaxel. No DLT was observed. The maximal administered dose of belinostat was 1000 mg m^−2^ per day for days 1–5, with paclitaxel (175 mg m^−2^) and carboplatin AUC 5 administered on day 3. Grade III/IV adverse events were (*n*; %): leucopenia (5; 22%), neutropenia (7; 30%), thrombocytopenia (3; 13%) anaemia (1; 4%), peripheral sensory neuropathy (*2;* 9%), fatigue (1; 4%), vomiting (1; 4%) and myalgia (1; 4%). The pharmacokinetics of belinostat, paclitaxel and carboplatin were unaltered by the concurrent administration. There were two partial responses (one rectal cancer and one pancreatic cancer). A third patient (mixed mullerian tumour of ovarian origin) showed a complete CA-125 response. In addition, six patients showed a stable disease lasting ⩾6 months.

**Conclusion:**

The combination was well tolerated, with no evidence of pharmacokinetic interaction. Further evaluation of anti-tumour activity is warranted.

Aberrant gene transcription is one of the hallmarks of cancer. Transcriptional regulation is controlled by an interaction of many processes including histone tail modification (Grunstein, 1997). The acetylation and deacetylation of histone tails is achieved by competing the activity of histone acetyl transferases and histone deacetylases (HDACs) (Grunstein, 1997). HDAC enzymes catalyse the removal of acetyl groups from lysine residues of histone proteins, compact chromatin and thus repress the transcription of associated genes. Deregulation in the expression of HDAC enzymes has been implicated in the development of cancers (Marks *et al*, 2001), and as such, HDAC enzymes represent a potential therapeutic target. HDAC inhibitors can induce apoptosis, cell cycle arrest and cellular differentiation, as well as inhibit angiogenesis (Marks and Dokmanovic, 2005; Bolden *et al*, 2006b). In addition, HDAC inhibitors have been shown to affect multiple pathways involved in oncogenesis through acetylation of non-histone proteins including heat shock protein-90, tubulin and hypoxia-inducible factor-1*α* (Bolden *et al*, 2006b; Schemies *et al*, 2009). A variety of HDAC inhibitors have already shown some anti-tumour activity in both pre-clinical and clinical settings (Bolden *et al*, 2006b).

Belinostat (PXD101) is a low molecular weight HDAC inhibitor of the hydroxamate class, having nanomolar activity against both class I and II recombinant HDAC isoforms with the advantage of both inducing histone acetylation and tubulin acetylation (Khan *et al*, 2008). It shows growth-inhibitory effects in a variety of human tumour cell lines *in vitro*, and human tumour xenografts *in vivo* (Plumb *et al*, 2003). This *in vitro* and *in vivo* growth inhibition was associated with a marked increase in the levels of acetylated histone proteins (Plumb *et al*, 2003). In addition, belinostat exhibited additive to synergistic when combined with paclitaxel, docetaxel and carboplatin both *in vitro* and *in vivo* (Qian *et al*, 2006), the postulated mechanism being through belinostat enhancing the acetylation of tubulin induced by docetaxel and phosphorylation of H2AX (variant of the histone H2A family) induced by carboplatin.

In two phase I studies of belinostat in solid (Steele *et al*, 2008) and haematological tumours (Gimsing *et al*, 2008), the treatment was well tolerated at the maximum tolerated dose (MTD) of 1000 mg m^−2^ per day administered intravenously (i.v.), q21 days. The dose-limiting toxicities (DLT) in the solid tumour study included fatigue, diarrhoea and atrial fibrillation, whereas none were seen in the study of haematological tumours. Importantly, no significant myelosuppression was observed.

On the basis of (i) the hypothesis that belinostat shows additive-to-synergistic activity when combined with commonly used cytototoxic agents including platinums (Pts) and taxanes and (ii) the lack of overlapping toxicity with these agents, this phase I study was designed to determine the MTD, DLT and the pharmacokinetic (PK) profile of belinostat, carboplatin and paclitaxel (BelCaP). Furthermore, it planned to explore the anti-tumour activity of the combination in solid tumours and in an MTD expansion of patients with relapsed advanced ovarian cancer.

## Materials and methods

### Eligibility

Patients with histological or cytological confirmation of advanced solid malignancy refractory to standard therapy were eligible, provided that they met the following criteria: age ⩾18 years; Eastern Co-operative Oncology Group performance status ⩽2; adequate bone marrow, hepatic and renal function (neutrophils >1.0 × 10^9^ l^−1^, platelets >100 × 10^9^ l^−1^, total bilirubin ⩽1.5 × upper limit of normal, aspartate aminotransferase, alanine aminotransferase, alkaline phosphatase ⩽3 × upper limit of normal (or ⩽5 × upper limit of normal if liver metastases) and calculated creatinine clearance ⩾45 ml min^−1^); female patients of reproductive potential were required to have a negative pregnancy test.

Patients were excluded if they had co-existing significant medical conditions including prolonged QTc >500 ms; use of concomitant medication that may cause torsade de Pointes; neuropathy ⩾grade II because of previous treatment; or had received >3 previous lines of chemotherapy for metastatic disease.

The study protocol was approved by the institutional ethics committee of the three sites participating in the study and all patients gave written informed consent before any study procedures were performed. The study was conducted in line with Good Clinical Practice in accordance with the Declaration of Helsinki and its amendments.

### Study design, objectives and treatment

This was an open-label phase I study with the primary objectives of determining the safety, DLT and MTD of BelCaP. Secondary objectives were to define the PK interaction between the agents as well as define preliminary anti-tumour activity.

Belinostat was administered in escalating doses of 600, 800 and 1000 mg m^−2^ per day as a 30-min i.v. infusion on days 1–5 q21. Carboplatin was administered as a 30-min i.v. infusion at area under the curve (AUC) 5 on day 3 q21 days, whereas paclitaxel was administered at 175 mg m^−2^ i.v. over 3 h. Cytotoxics were given 2–3 h after the administration of belinostat–paclitaxel followed by carboplatin. This schedule of belinostat followed by cytotoxics was chosen on the basis of pre-clinical *in vitro* and *in vivo* data supporting a higher degree of synergy, when HDAC inhibitors were administered before topoisomerase II therapy (Marchion *et al*, 2004, 2005). In addition, 48 h pre-exposure with belinostat was shown to be better than 24 h, which was better than 12 h. Carboplatin was dosed in accordance with the Calvert formula using ethylenediaminetetraacetic acid clearance to estimate glomerular filtration rate (GFR) (Calvert *et al*, 1989) at the European Union sites and the Jelliffe formula at sites in the United States (Jelliffe, 1973). Prophylactic anti-emetic and hypersensivity prophylaxis for paclitaxel were administered as per local standards.

A DLT was defined as an absolute neutrophil count <0.5 × 10^9^ l^−1^ lasting for ⩾7 days, or absolute neutrophil count <0.5 × 10^9^ l^−1^ with sepsis; or platelet count <25 × 10^9^ l^−1^. In addition, any other drug-related non-haematological grade III or IV toxicity with the exceptions of alopecia, nausea and vomiting, diarrhoea, rash, arthralgias and myalgias was categorized as DLT. Treatment interventions were attempted to palliate toxicity symptoms before concluding a DLT had occurred. The patients who experienced DLT with documented clinical benefit (stable disease (s.d.) or an objective response) were allowed to continue treatment with BelCaP at the before dose level, if toxicity had resolved to less than grade II (except for alopecia).

Dose escalation was planned in a classical 3+3 design in up to five cohorts. In cohort 1A, belinostat was to be administered at 600 mg m^−2^ together with carboplatin AUC 5; in cohort 1B, paclitaxel was to be evaluated with paclitaxel 175 mg m^−2^. In cohort 2, belinostat at 600 mg m^−2^ was to be administered with both carboplatin AUC 5 and paclitaxel at 175 mg m^−2^. In cohorts 3 and 4, belinostat was to be administered at 800 and 1000 mg m^−2^, respectively, together with the same doses of BelCaP. Treatment cycles were to be repeated until disease progression, evidence of significant treatment-related toxicities, or withdrawal of consent. Up to two dose reductions were to be allowed of all three drugs. The patients who did not complete the first cycle were replaced.

### Study assessments

Pre-treatment evaluation included history, physical examination, laboratory assessments, chest x-ray, electrocardiogram (ECG), urinalysis and tumour evaluation with computed tomography (CT) or magnetic resonance imaging (MRI). Laboratory assessments (complete blood count, clotting screen, urea, creatinine, electrolytes, liver function tests, uric acid, glucose and cholesterol) were performed pre-treatment and weekly thereafter as well as on day 4 of cycles 1 and 2 only. Electrocardiograms were performed before and within 1 h after treatment with belinostat in cycle 1 and on day 1 only of all subsequent cycles. Central ECG reading was carried out using a digital analysis method.

Toxicities were graded according to the National Cancer Institute Common Terminology Criteria for Adverse Events version 3.0. Efficacy was assessed by CT or MRI performed every two cycles using Response Evaluation Criteria in Solid Tumors (RECIST) (Therasse *et al*, 2000).

### Drug preparation

Belinostat was supplied by Topotarget as 10-ml vials, each containing 50 mg ml^−1^ belinostat and the solubilizer L-arginine (100 mg ml^−1^). Belinostat was stored below 5°C. The assigned dose of belinostat was added to a 250-ml bag of sterile sodium chloride (0.9% solution or 5% dextrose solution) and immediately used. Carboplatin and paclitaxel were prepared as indicated in the summary of product characteristics for each agent.

### Pharmacokinetic sampling and analysis

Blood samples for belinostat PK, when administered alone were taken pre-treatment, at the end of infusion and 5 min, 15, 30 min, 1 h, 2, 3 h, 3 h 15 min, 3 h 30 min, 4, 5, 6 h after infusion on days 1 and 3 of cycle 1. Samples for carboplatin or paclitaxel when administered after belinostat, were taken relative to the start of belinostat infusion at 3 h (pre-carboplatin or-paclitaxel), 3 h 15 min, 3 h 30 min, 4, 5 and 6 h in cycle 1 on day 3. In addition, samples were taken for BelCaP when administered together at 6 h 15 min, 6 h 30 min, 7, 8, 9 and 24 h after belinostat on day 3. Belinostat and paclitaxel levels were determined in lithium-heparinised plasma using validated solid-phase extraction high performance liquid chromatograpgy (HPLC)-tandem mass spectrometric detection (MS/MS) methods (Covance, Indianapolis, IN, USA). The lower limit of quantitation was 5 and 10 ng ml^−1^ for belinostat and paclitaxel, respectively. Belinostat plasma concentrations were determined at Covance using a validated method involving solid-phase extraction, separation with acetic acid acetonitril gradient on reversed phase C18 silica and MS/MS using penta-deuterated belinostat as the internal standard. Belinostat and the internal standard were detected using negative ion electron spray with the transition from 317 to 143 and 322 to 143 m z^−1^, respectively.

Paclitaxel and the internal standard, cephalomannine, are extracted from human plasma by solid-phase extraction on a cyanopropyl solid-phase extraction cartridge. After eluting with the mobile phase, the eluent is directly analysed using liquid chromatography with MS/MS. The standard curve range for this assay is from 10.0 to 2000 ng ml^−1^ for paclitaxel, using a plasma sample volume of 0.250 ml.

Human plasma samples were analysed for Pt from carboplatin by inductively coupled plasma-mass spectrometry after dilution with 0.01% Triton X-100 in 0.1% nitric acid. The samples were analysed without pre-digestion or extraction directly against matrix-matched standard solutions. Intensity, as counts per second from Pt, mass 195, was measured. Terbium, mass 159, was used as an internal standard. The results were calculated from the net intensity ratio of Pt to internal standard. The standard curve range was 2.00–1000 ng Pt ml^−1^ in plasma. Samples above the calibration range were diluted using blank human plasma.

Plasma concentration–time curves of all three analytes were analysed by non-compartmental methods using WinNonlin (version 4.0.1 Pharsight Corp., Cary, NC, USA). For all analytes the AUC was estimated from the start of infusion by the logarithmic-linear trapezoidal rule and extrapolated to infinity using the estimated value of the slope of the terminal logarithmic-linear disposition phase. Analysis was carried out on the non-dose-normalised plasma concentration of carboplatin, as dosing had already been normalised per GFR using Calvert's formula (Calvert *et al*, 1989).

## Results

### Patient characteristics

In all 23 patients with a median age of 56 years (range 39–66) were enroled between August 2005 and August 2006; the demographics are described on Table 1. A total of 139 complete cycles were administered (median 4; range 0–32; Table 2). A total of 10 patients completed ⩾6 cycles of treatment.

### Safety

All patients were evaluable for safety. Dose escalation proceeded through all cohorts with no observed DLT attributable to the combination of BelCaP. There were no grade IV non-haematological adverse events (AE). Grade III AEs observed were peripheral sensory neuropathy (*n*=2), fatigue (*n*=1), vomiting (*n*=1) and myalgia (*n*=1) (Table 3). There was no cardiac toxicity; two events of investigator-reported QTc prolongation in two patients were later refuted by central ECG analysis. Grade III haematological AEs comprised (*n*) leucopenia (5), neutropenia (3), thrombocytopenia (3) and anaemia (1e) (Table 3). Three patients experienced grade IV neutropenia. There was no evidence that the incidence of haematological AEs increased with the addition of belinostat to BelCaP. As per the protocol no further dose escalation beyond 1000 mg m^−2^ per day was attempted, and the recommended dose was defined as belinostat (1000 mg m^−2^ per day) by 30-min i.v. infusion once daily for 5 days every 21 days, with paclitaxel (175 mg m^−2^) and carboplatin AUC 5 administered on day 3.

### Pharmacokinetics

The belinostat pharmacokinetics was dose proportional at the dose range evaluated (Table 4). In five of seven patients, the pre-infusion values on day 3 were below the limit of quantitation, indicating that there was no accumulation of belinostat at the 1000 mg m^−2^ per day level. Administration of BelCaP on day 3 did not alter the PK of belinostat. Similarly, there was no effect of increasing the belinostat dose on the PK parameters of either paclitaxel or carboplatin and these were comparable with that reported in the literature (Tables 5 and 6) (Oguri *et al*, 1988; Bhalla *et al*, 1999; Goffin *et al*, 2005).

### Efficacy

**Figure 1:**
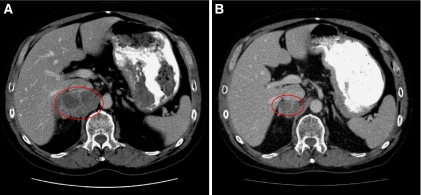
Efficacy of BelCaP in a patient with rectal cancer. Response in a patient with metastatic rectal carcinoma treated with BelCaP showing an adrenal metastasis at baseline, (**A**) (encircled in red) measuring 7.7 cm, and after six cycles, (**B**) measuring 3.6 cm. The patient also had retroperitoneal lymph node metastases and with this, showed a confirmed PR. (See online version for color information).

In all 14 patients completed at least two cycles and were evaluable for tumour response. Two patients showed a confirmed partial response (PR), including one patient with metastatic pancreatic cancer treated with belinostat and carboplatin and the other patient with metastatic rectal cancer treated with belinostat with BelCaP. The duration of response was 7 and 9 months, respectively. Figure 1 shows the reduction in the size of adrenal metastases in the patient with metastatic rectal carcinoma. These patients had not previously received carboplatin or paclitaxel, but the latter patient had received oxaliplatin. A third patient with a mixed mullerian ovarian tumour, with radiologically s.d. by RECIST, showed a complete CA-125 response. This patient had previously received both BelCaP. A fourth patient with metastatic transitional cell cancer of the bladder bones only, treated with belinostat and paclitaxel, showed a resolution of a bony metastatic deposit, but was otherwise rated s.d. by investigator. This patient was previously treated with carboplatin and gemcitabine with a best response of progressive disease.

**Figure 2:**
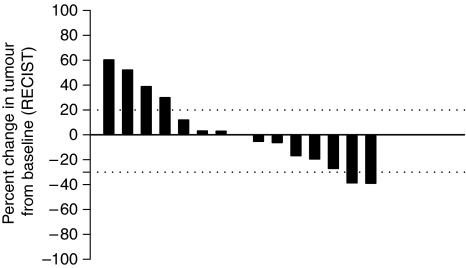
Waterfall plot showing best response in individual patients evaluable for responses assessment by RECIST 1.0 criteria. In all14 patients completed the first two cycles and were evaluable for response and featured in this plot. Dotted lines at +20% and −30% indicate the % level at which disease progression and disease response has occurred, respectively.

Overall, eight patients showed a best response of s.d. (median 6.6 months; range 1–29); in seven of these eight patients s.d. lasted ⩾ 6 months (range 6–29 months). These included patients with metastatic transitional cell carcinoma (TCC) bladder, metastatic cholangiocarcinoma, carcinoma of unknown primary (CUP), ovarian cancer, rectal cancer, mesothelioma and Ewing sarcoma. Five of these six patients had received Pt-based treatments previously. Figure 2 shows a waterfall plot of the 14 patients evaluable for response.

## Discussion

The safety and MTD of belinostat have been previously described (Steele *et al*, 2008). We now present data from a phase I trial of a first-in-man combination of the HDAC inhibitor with carboplatin and/or paclitaxel, BelCaP. Carboplatin and paclitaxel were well tolerated with no evidence of DLT up to the maximum administered dose of 1000 mg m^−2^ per day belinostat with BelCaP at standard doses. The most common toxicities were gastrointestinal and constitutional, often associated with BelCaP alone and seen with single agent belinostat. However, there was no evidence that the incidence or severity of these, or of haematological toxicity, increased with the combination. It should be noted that there was no evidence of significant cardiac toxicity, which has been associated with HDAC inhibitors.

The toxicity profile of BelCaP was favourable and grade III/IV neutropenia was reported in 26% of the patients and this was probably attributed to BelCaP. Ramalingan *et al* reported that the combination of the HDAC inhibitor vorinostat with BelCaP, resulted in grade III/IV neutropenia in 50% of the patients, but in their study the dose of BelCaP was higher than in the present study (Ramalingam *et al*, 2007a). Furthermore, when valproic acid was combined with epirubicin, neurological DLTs were attributed to valproate with no exacerbation of epirubicin-related myelosuppression (Munster *et al*, 2007). However, grade II–III QTc prolongation was seen in approximately 25% of patients in that study and as previously reported, this was, in nearly all cases, associated with electrolyte abnormalities (Munster *et al*, 2007; Piekarz *et al*, 2006).

PK results for belinostat showed that there was a dose-proportional increase in belinostat exposure with increasing dose and that there was no accumulation with repeat dosing. There seemed to be no significant change in the PK parameters of belinostat with the addition of BelCaP when compared with the PK parameters seen in the single-agent phase I study (Steele *et al*, 2008). The similarity between the PK parameters of BelCaP in this study with historical controls, suggests that this combination is feasible, although such a comparison with historical data has its limitations.

Encouragingly, BelCaP showed evidence of clinical activity in heavily pre-treated patients, including those previously exposed to Pt therapy. As a result of the clinical benefit in a patient with metastatic TC of the bladder to bone, an expansion cohort of BelCaP was initiated in patients with this tumour type.

Clinical efficacy has been observed with other combinations of an HDAC inhibitor and cytotoxic chemotherapy. A total of 9 of 41 patients, with a variety of solid tumours, including prostate, pancreatic, melanoma and cervical cancer, responded to valproic acid with epirubicin; in a phase II portion of this study, 64% of patients with breast cancer responded to treatment (Munster *et al*, 2009). In the study of Ramalingam *et al* of 25 patients treated with vorinostat and BelCaP, 10 patients with non-small-cell lung and 1 patient with head and neck cancer showed a PR (Ramalingam *et al*, 2007a). However, the majority of patients (82%) was chemotherapy naive. The efficacy of this belinostat combination fares favourably and merits further evaluation.

In summary, the combination of belinostat (100 mg m^2^ per day) with carboplatin AUC5 and paclitaxel (175 mg m^−2^) is well tolerated, with no evidence of any PK interaction. Recruitment of patients to two expansion cohorts of ovarian cancer and TCC of bladder has recently been completed and an evaluation in patients with CUP is ongoing.

## Figures and Tables

**Table 1:** Patient characteristics

**Characteristic**	**No. of patients**
Total	23
	
*Age (years)*	
Median	56
Range	39–66
	
Sex	
Male	14
Female	9
	
*ECOG performance status*	
0	14
1	7
2	2
	
*Previous anti-tumour regimens*	
0	1
1	8
2	4
3	7
4	3
	
*Primary tumour type*	
Pancreatic carcinoma	3
Osteo/chondrosarcoma	3
Soft tissue sarcoma	2
Rectal carcinoma	2
Gastric carcinoma	2
Carcinoma of unknown primary	2
Ovarian carcinoma	2
Malignant melanoma	2
Mesothelioma	2
Bladder carcinoma	1
Hepatocellular carcinoma	1
Cholangiocarcinoma	1

Abbreviation: ECOG=Eastern Co-operative Oncology Group.

**Table 2:** Dose escalation, exposure and dose-limiting toxicity

**Cohort**	**Belinostat day 1–5 (mg m^−2^)**	**Carboplatin day 3 (AUC^a^)**	**Paclitaxel day 3 (mg m^−2^)**	**No. of patients^b^**	**Total no. of completed cycles**	**Median no. of completed cycles (range)**	**Dose-limiting toxicity**
1A	600	5	—	5	25	4 (1–12)	—
1B	600	—	175	5	40	6 (0–20)	—
2	600	5	175	3	9	2 (1–6)	—
3	800	5	175	4	36	2 (0–32)	—
4	1000	5	175	6	29	5 (2–9)	—

Abbreviations: AUC=area under curve; EDTA=ethylenediaminetetraacetic acid; GFR=glomerular filtration rate.

a Calvert formula using chrome-EDTA clearance to estimate GFR.

b Patients who did not complete the first cycle were replaced.

**Table 3:** Non-haematological and haematological treatment related adverse events

	**Cohort, *n***					
**Event or grade**	**1A**	**1B**	**2**	**3**	**4**	**Total (%)**
*Non-haematological*						
Nausea						
1–2	2	3	1	3	4	13 (57)
3	0	0	0	0	0	0
Fatigue						
1–2	1	4	1	2	3	11 (48)
3	0	0	0	1	0	1 (4)
Vomiting						
1–2	1	1	0	3	3	8 (35)
3	0	0	1	0	0	1 (4)
Peripheral sensory neuropathy						
1–2	0	3	0	1	2	6 (26)
3	0	0	1	1	0	2 (9)
Alopecia						
1–2	0	3	1	2	2	8 (35)
3	0	0	0	0	0	0
Flushing						
1–2	2	3	0	2	1	8 (35)
3	0	0	0	0	0	0
Myalgia						
1–2	0	3	1	1	2	7 (30)
3	0	0	1	0	0	1 (4)
Arthralgia						
1–2	0	3	1	1	1	6 (26)
3	0	0	0	0	0	0
						
*Haematological*						
Anaemia						
1–2	5	4	2	3	3	17 (96)
3	0	0	0	0	1	1 (13)
Leucopenia						
1–2	2	4	1	3	2	12 (61)
3	0	0	0	3	2	5 (22)
Neutropenia						
1–2	2	1	2	0	6	11 (65)
3	1	0	0	2	0	3 (13)
4	0	0	0	1	2	3 (13)
Thrombocytopenia						
1–2	1	2	1	1	1	6 (48)
3	1	0	0	1	1	3 (13)

**Table 4:** Pharmacokinetic variables for belinostat (mean±s.d. for a single i.v. dose on days 1 and 3)

**Belinostat dose (mg m^−2^)**	**No. of patients**	***T*_1/2_ (h)**	***C*_max_ (*μ*M)**	**AUC_all_ (h *μ*M)**	**AUC_inf_ (h *μ*M)**	**Cl** **(l h^−1^ m^−2^)**	**Vss** **(l m^−2^)**
600	12	1.7±0.9	58.5±13.8	28.4±7.1	28.4±7.1	69.7±15.2	34.9±6.4
800	4	1.7±0.2	70.8±18.9	35.5±8.7	35.6±8.7	73.7±14.1	38.1±9.7
1000 day 1	7	1.0±0.1	127.6±43.5	101.9±58.2	102.0±58.3	41.1±22.2	22.6±9.7
1000 day 3	7	2.7±0.4	134.7±54.9	88.8±63.9	88.8±63.9	45.1±17.3	35.8±17.3

Abbreviations: AUC=area under curve; Cl=clearance; i.v.=intravenous; Vss=steady state volumes of distribution.

**Table 5:** Pharmacokinetic variables for paclitaxel (mean±s.d. for a single i.v. dose of 175 mg m^−2^ on day 3 after belinostat administration)

**Belinostat dose (mg m^−2^)**	**No. of patients**	***T*_1/2_ (h)**	***C*_max_ (*μ*M)**	**AUC_all_ (h *μ*M)**	**AUC_inf_ (h *μ*M)**	**Cl (l h^−1^ m^−2^)**	**Vss (l m^−2^)**
600	6	5.7±0.5	3.9±0.7	14.0±2.5	14.7±2.6	14.3±2.2	56.6±21.2
800	3	5.5±1.1	3.9±0.7	12.8±1.7	13.3±2.0	15.7±2.6	53.0±3.0
1000	7	5.5±0.6	3.9±1.3	14.4±3.8	15.0±4.0	14.8±4.9	57.2±31.7

Abbreviations: AUC=area under curve; CV=cardiovascular; i.v.=intravenous.

**Table 6:** Pharmacokinetic variables for Carboplatin equivalents^a^ (mean±s.d. for a single i.v. dose with target AUC 5 on day 3 after belinostat and paclitaxel administration)

**Belinostat dose (mg m^−2^)**	**No. of patients**	**Carboplatin dose (mg m^−2^)**	***T*_1/2_ (h)**	***C*_max_ (*μ*g ml^−1^)**	**AUC_all_ (mg·min ml^−1^)**	**AUC_inf_ (mg·min ml^−1^)**	**Cl (l h^−1^)**	**Vss (l)**
600	7	630.0±98.6	7.8±2.7	27.4±4.0	7.8±1.4	8.8±1.3	4.4±0.9	34.3±16.1
800	3	655.0±138.1	5.4±1.0	32.8±11.7	8.9±2.1	9.7±1.9	4.1±0.5	21.9±7.6
1000	7	537.1±105.4	6.0±0.8	29.6±14.0	8.4±1.0	9.3±1.1	3.5±0.8	20.4±3.5
Total Mean		596.2±113.4	6.6±2.1	29.2±1.0	8.2±1.4	9.2±1.3	3.9±0.8	26.4±12.5
%CV		19.0	31.0	34.1	17.0	14.2	21.4	47.3

Abbreviations: AUC=area under curve; Cl=clearance; CV=cardiovascular; i.v.=intravenous; Vss=steady state volumes of distribution.

a Total platinum measured was converted to carboplatin equivalent by use of a 0.5254 conversion factor corresponding to ratio of formula weights; platinum 195.078 g mol^−1^ and carboplatin 371.3 g mol^−1^.
